# Follicle Loss and Apoptosis in Cyclophosphamide-Treated Mice: What’s the Matter?

**DOI:** 10.3390/ijms17060836

**Published:** 2016-05-30

**Authors:** Xiu-Ying Chen, He-Xia Xia, Hai-Yun Guan, Bin Li, Wei Zhang

**Affiliations:** 1 Obstetrics and Gynecology Hospital, Fudan University, Shanghai 200011, China; chenxiuying36@163.com (X.-Y.C.); xhx0101@hotmail.com (H.-X.X.); 15211250003@fudan.edu.cn (H.-Y.G.); libin782@126.com (B.L.); 2 Department of Obstetrics and Gynecology of Shanghai Medical College, Fudan University, Shanghai 200011, China; 3 Shanghai Key Laboratory of Female Reproductive Endocrine Related Diseases, Shanghai 200011, China

**Keywords:** cyclophosphamide, follicle, signaling pathway

## Abstract

With increasing numbers of young female cancer survivors following chemotherapy, chemotherapy-induced fertility loss must be considered. Menstrual disorder and infertility are of particular concern in female cancer patients. We showed that treatment with the alkylating agent cyclophosphamide (CTX) could cause severe primordial follicle loss and growing follicle apoptosis, resulting in loss of ovarian reserve. SPF C57BL/6 female mice were treated with a single dose of 120 mg/kg of CTX or saline as a control, and both sides of ovaries were collected three or seven days after injection. Following CTX treatment, the ovaries were mostly composed of collapsed oocytes and presented marked cortical fibrosis and a reduced number of follicles, especially primordial follicles. The loss of primordial follicles was confirmed by primordial follicle counting, immunohistochemistry and Western blot detection of DDx4/MVH. Follicle apoptosis was tested by a TUNEL assay and the number of TUNEL-positive follicle cells increased, as expected, in CTX-treated mice. Furthermore, expression of APAF-1 and cleaved caspase-3 was also increased after CTX treatment. Analysis of the PI3K/Akt/mTOR signaling pathway showed that CTX increased phosphorylation of Akt, mTOR and downstream proteins without affecting total levels. These results demonstrated that the CTX treatment led to the hyperactivation of the PI3K/Akt/mTOR signaling pathway in ovaries which may be related to primordial follicle loss and growing follicle apoptosis.

## 1. Introduction

With the advances in the early diagnosis and effective treatment of many cancer patients, long-time survival has become a reality for many young cancer patients during their reproductive years [1]. However, a great majority of cancer survivors are at risk of fertility loss due to premature ovarian insufficiency (POI) following aggressive cytotoxic chemotherapy drugs [2,3]. Chemotherapy-induced ovarian failure acts mainly through the prevention of cell division and the inhibition of DNA function, which could induce apoptosis in pre-granulosa cells and damage ovarian stromal components, resulting in the reversible premature exhaustion of the resting primordial follicles [4]. Previous studies have shown that alkylating agents could cause cortical fibrosis, blood vessel damage, and severe reduction in primordial follicles (which is presumed to interrupt essential cell processes) [5]; these agents could also arrest cell proliferation and cause apoptosis in granulosa cells [6]. Cyclophosphamide (CTX), one such alkylating agent, is commonly used in the treatment of many types of malignant tumors such as Hodgkin’s disease and breast cancer [7,8]. In spite of many beneficial effects due to CTX application, several adverse reactions and side effects of CTX have been reported. CTX treatment could cause injuries to normal tissue, such as peroxidative damage to the kidneys, acute cardiotoxic effects and bone marrow suppression [9,10]. CTX could induce cytotoxicity by creating DNA cross-links and promoting DNA breaks [11], and follicular decline in CTX-treated ovaries may occur in a dose-dependent manner [12]. Despite much effort to understand the details of CTX-induced ovary damage and insufficiency, the molecular mechanisms are still unclear. Therefore, the purpose of our study is to examine the effects of CTX on ovaries and investigate which signaling pathway participates in ovarian injury due to CTX treatment.

## 2. Results

### 2.1. CyclophosphamideInduced Primordial Follicle Loss in Female Mice

**Figure 1:**
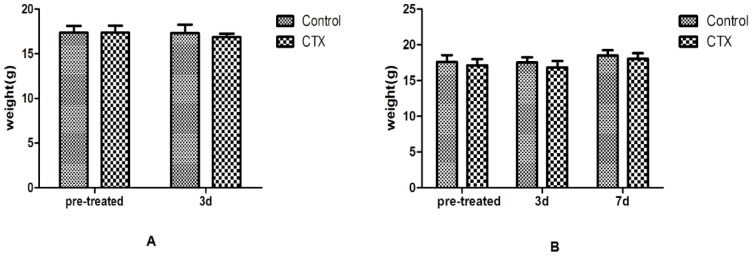
A single dose of Cyclophosphamide injection did not change body weight at different time points. (**A**) Three days after CTX treatment; (**B**) Seven days after CTX treatment. The results show the means ± SD.

**Figure 2:**
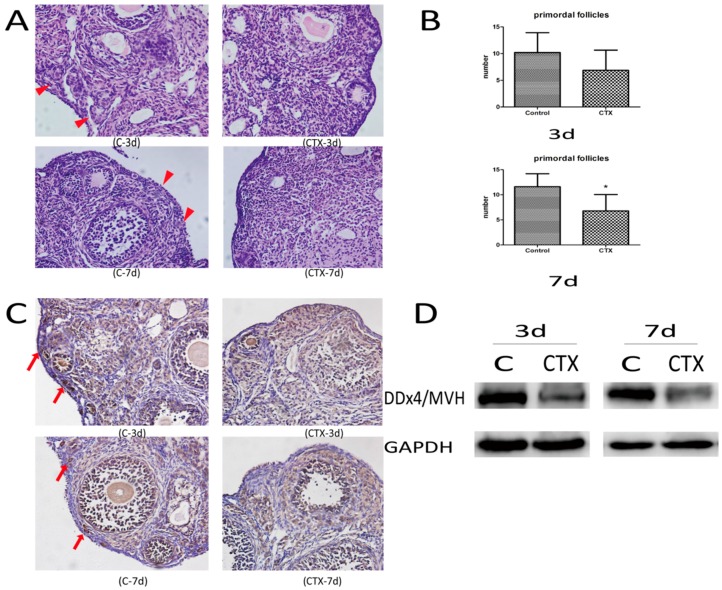
Cyclophosphamide induced primordial follicles loss in female mice. (**A**) Representative images of H&E dyeing for ovarian histomorphology (original magnification 400×), the red triangle indicates primordial follicles; (**B**) Primordial follicle counts after CTX treatment, and the whole fields of view were counted and the average of three sections was calculated in one mouse. The graph shows the means ± SD, * *p* ≤ 0.05 (Independent-Samples *t*-test); (**C**) Representative images of DDx4/MVH staining for primordial germ cells (original magnification 400×), the red arrow indicates dyeing primordial follicles; (**D**) DDx4/MVH expression detected by Western blot. More than three ovarian samples of each group were analyzed and we chose the representative images in the figure.

There was no death and no apparent toxicity in mice after CTX injection, and body-weight gains were normal (Figure 1). The ovaries from control groups presented large numbers of follicles at different stages. Although follicles at different stages could be observed in CTX-treated mice, the ovaries were mostly composed of collapsed oocytes and presented marked cortical fibrosis and a reduced number of follicles, especially primordial follicles (Figure 2A). Primordial follicle counts showed a significant reduction of primordial follicles in the ovaries treated with CTX (Figure 2B), with this reduction being more obvious seven days after CTX treatment. Immunohistochemical (Figure 2C) and Western blot (Figure 2D) assays also showed that DDx4/MVH expression was markedly lower after CTX treatment than in the control group.

### 2.2. Cyclophosphamide Accelerated Growing Follicle Apoptosis

**Figure 3:**
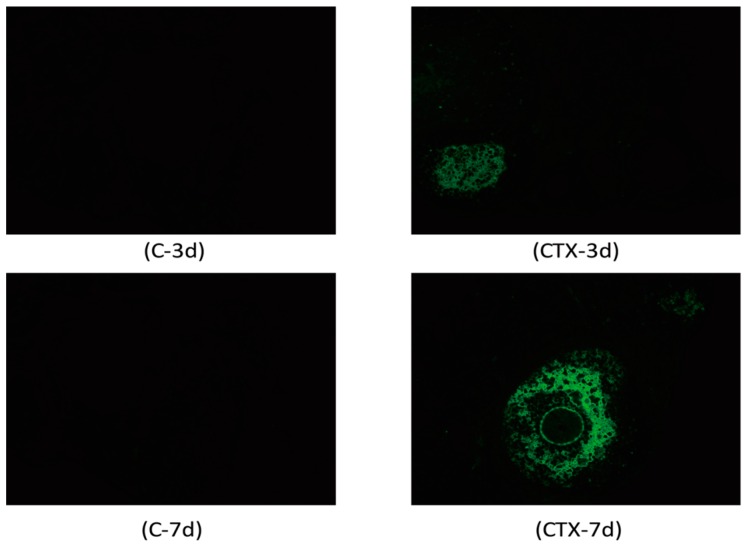
Cyclophosphamide accelerated growing follicle apoptosis: representative images of TUNEL staining for follicle cell apoptosis on ovaries. Green fluorescence indicates apoptotic follicle cells (original magnification 200×).

**Figure 4:**
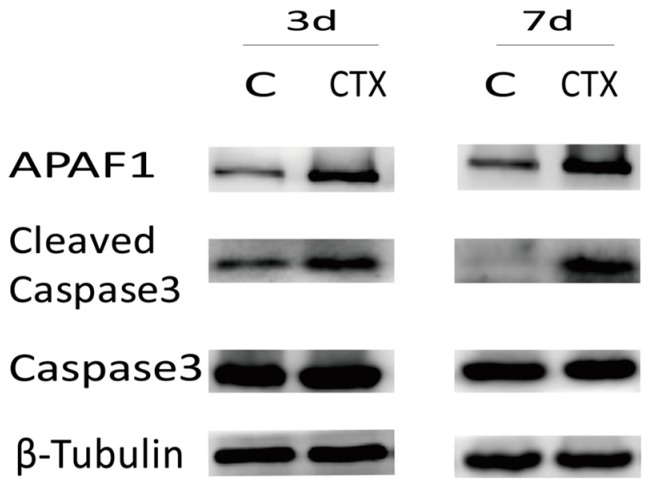
Cyclophosphamide activated the classical apoptosis signaling pathway: expression of APAF-1, cleaved caspase-3 and total caspase-3. More than three ovarian samples of each group were analyzed by Western blot and we chose the representative images in the figure.

To identify cleavage of genomic DNA strand breaks during apoptosis, free 3’-OH termini could be labeled with modified nucleotides in an enzymatic reaction. This TUNEL technology could detect and quantify apoptosis at the single cell level. TUNEL staining of a paraffin section of ovaries showed no apparent apoptosis in primordial follicles of either the control or CTX-treated mice. However, a large number of TUNEL-positive cells were observed in granulosa cells of the growing follicle populations in CTX-treated ovaries (select images are shown in Figure 3). These cells were scattered apoptotic cells at normal physiological levels similar to the ones seen in control groups. Moreover, APAF-1 and cleaved caspase-3 levels were elevated after CTX treatment, but the total caspase-3 levels did not change (Figure 4). These results demonstrated that CTX treatment leads to the hyperactivation of the apoptosis signaling pathway in ovaries.

### 2.3. The PI3K/Akt/mTOR Signaling Pathway May Participate in Ovarian Injury Due to CTX Treatment

**Figure 5:**
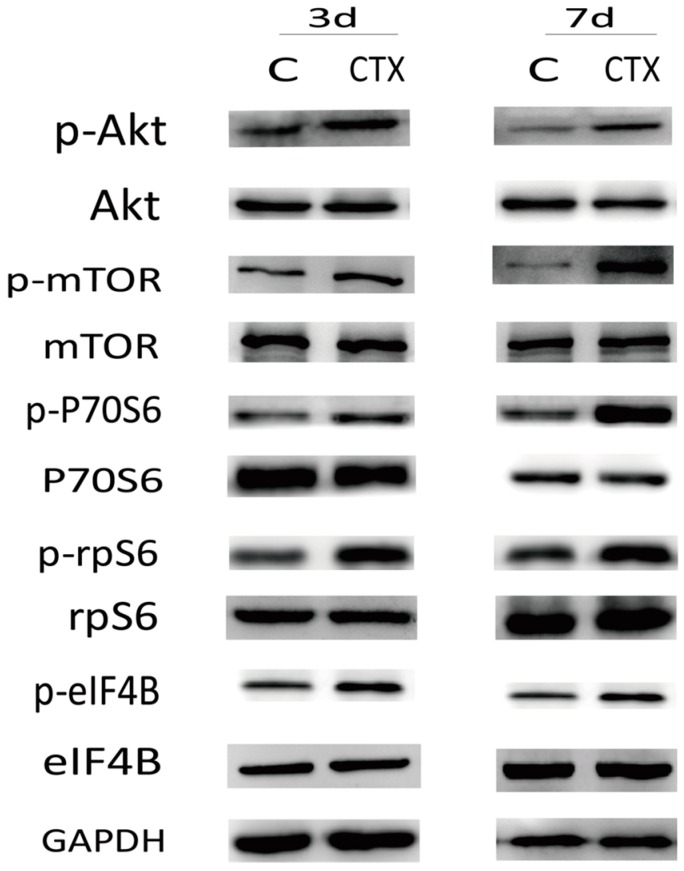
Cyclophosphamide induced increased phosphorylation of PI3K/Akt/mTOR signaling pathway proteins in the ovary: expression of Akt, mTOR, and the downstream P70S6-rpS6-eIF4B proteins. More than three ovarian samples of each group were analyzed by Western blot and we chose the representative images in the figure.

We find that the mouse ovaries undergo primordial follicle loss and growing follicle apoptosis after being treated with CTX. Additionally, the PI3K/Akt/mTOR signaling pathway regulates the activation of primordial follicles. So, is this signaling pathway participating in ovarian injury due to CTX treatment? To explore whether the PI3K/Akt/mTOR signaling pathway could be involved in ovarian injury due to CTX treatment, we investigated ovarian phosphorylation of Akt, mTOR and its downstream molecules after CTX treatment. As shown in Figure 5, the ovaries from CTX-treated mice showed increased phospho-Akt and phospho-mTOR levels without affecting the total mTOR content. We further monitored the phosphorylation of the downstream proteins P70S6, rpS6 and eIF4B in ovarian tissues. CTX treatment also increased the phosphorylation of these downstream proteins without affecting total levels. These findings demonstrated that the PI3K/Akt/mTOR signaling pathway in the ovary was over-activated by CTX treatment.

## 3. Discussion

Primordial follicles are the most important subset of follicles because the number of primordial follicles is fixed before birth and cannot be renewed. During each menstrual period, a limited number of primordial follicles are recruited and develop into growing follicles, and the balance of quiescence and activation of primordial follicles determines the length of a female’s reproductive life [13]. The activation of primordial follicles is a highly regulated process, and the depletion of the primordial follicle stockpile is irreversible. Any factors that alter this balance may result in an accelerated depletion of primordial follicles and, ultimately, ovarian failure. The molecular mechanisms underlying the activation and development of primordial follicles have begun to be elucidated in recent years. Using genetically modified mouse models, previous researchers have shown that an enhanced PI3K or mTOR signaling pathway was involved in follicular activation [14]. The functional role of PI3K signaling was enhanced when *Pten (phosphatase and tensinhomolog deleted on chromosome ten)*, a negative regulator of PI3K, was deleted specifically from oocytes, which resulted in the global activation of oocytes and the exhaustion of the entire primordial follicle pool [15]. Loss of the *Pten* gene resulted in an enhanced PI3K signaling pathway which was indicated by an increase in phosphorylated Akt (p-Akt). Akt is known to mediate the activation of the mammalian target of rapamycin complex 1 (mTORC1) through multiple mechanisms, as an upstream regulator of mTORC1 [16]. By deleting *Tsc1* specifically in mice oocytes, studies have also shown that increased activation of mTORC1 in mouse oocytes led to the premature activation and depletion of primordial follicles [17]. The activation of P70S6–rpS6 signaling was enhanced by elevated mTORC1 activity, which promoted protein translation and ribosomal biogenesis inoocytes [18]. These *Pten*- or *Tsc1*-deficientmice showed an identical phenotype with untimely activation of primordial follicles and eventual exhaustion through elevated levels of phosphorylated-Akt, mTOR and downstream proteins. Together, these studies suggested that the PI3K/Akt/mTOR signaling pathway regulates the activation of primordial follicles.

CTX is commonly used as an effective treatment for malignancies and autoimmune disease, and an assistant strategy for transplantation. Additionally, CTX is a prodrug activated mainly by cytochrome P450 and metabolized to the active metabolite 4-hydroxy-cyclophosphamide (4-OH-Cy) in liver [19]. CTX could seriously damage ovarian endocrine function and induce fertility due to its gonadal toxicity. This *in vivo* mouse study demonstrates that CTX severely destroyed the dormant primordial follicle reserve as evidenced by the follicle counts conducted three and seven days after CTX treatment. Additionally, as shown in the histomorphology images, the ovaries were mostly composed of atretic or collapsed oocytes and presented marked cortical fibrosis and a reduced number of follicles, especially primordial follicles. Anti-DDX4/MVH antibody was used as a primordial germ cell marker to further confirm that CTX administration did the most harm to primordial follicle reserve, which was also independently verified by both immunohistochemistry and Western blot detection. Furthermore, we tried to detect follicle apoptosis via an *in situ* cell death detection kit as an *in situ* method by detecting areas of DNA that are nicked during apoptosis, and obvious follicle apoptosis was observed in CTX-treated mice. The classical apoptosis pathway was also activated by increased expression of APAF-1 and cleaved caspase-3 after CTX treatment. APAF-1containing an amino-terminal CARD domain, a central CED-4 homology domain, and multiple WD-40 repeats at the carboxy-terminus was an important signaling protein involved in the apoptosis pathway, which led to caspase-9 activation and subsequent caspase-3 activation. Caspase-3 was a critical apoptosis trigger, with cleavage of caspase-3 requiring the aspartic acid residue at the P1 position. In this study, APAF-1 and cleaved caspase-3 expression was elevated after CTX treatment, which indicated that this apoptosis pathway was activated. Consistent with previous studies, these results show that CTX treatment induced a wave of primordial follicle loss and growing follicle apoptosis. However, what is the underlying molecular mechanism?

In this study, activation of the PI3K/Akt/mTOR pathway in ovaries of CTX-treated mice was demonstrated by increased phosphorylation of Akt, mTOR, and the downstream P70S6-rpS6-eIF4B proteins. CTX treatment enhanced the phosphorylation of Akt and mTOR, and resulted in activation of P70S6-rpS6-eIF4B signaling in oocytes, which may be the reason for the rapid primordial follicle depletion. The activation and development of primordial follicles and the apoptosis of most growing follicles are progressive and highly regulated processes. The initial size of the primordial follicle pool and the rate of its activation and depletion determine the duration of female fertility. Exposure to CTX disturbed this balance via up-regulation of the PI3K/Akt/mTOR pathway, which induced excessive primordial follicle activation and caused growing follicles to undergo apoptosis. Finally, primordial follicles were recruited into a vicious cycle of growth, development, and death, which causes the reservoir to exhaust.

## 4. Materials and Methods

### 4.1. Mice

SPF C57BL/6 female mice (five weeks of age) were obtained from Shanghai Slack Laboratory Animal Co., Ltd. (Shanghai, China). All experimental mice were housed in groups of four per wire cage and kept under standard laboratory conditions (12 h of light, 12 h of dark; 25 °C). All animal experiments were approved by the Experimental Animal Ethical Committee of Fudan University. (Approval No.:2012-36, Approval Date: 20 February 2012) After acclimatizing, 40 mice were divided into four groups. Groups A (C-3d) and B (C-7d) received a single intraperitoneal injection of saline. Groups C (CTX-3d) and D (CTX-7d) were treated with a single dose of 120 mg/kg of CTX (Sigma Aldrich, St. Louis, MO, USA) by intraperitoneal injection. Groups A and C mice were sacrificed three days after treatment, and Group B and D mice were sacrificed seven days after treatment. Both sides of the ovaries were collected.

### 4.2. Ovarian Histomorphology

Ovaries from the left side of each mouse were fixed in 4% paraformaldehyde for 48 h and then dehydrated and embedded in paraffin. The paraffin-embedded ovaries were serially sectioned at 4mm thickness. The sections were then rehydrated and stained in hematoxylin and eosin for histomorphological observation and primordial follicle count.

### 4.3. Immunohistochemical Analysis

The sections was incubated in 0.01 M citrate buffer (pH 6.0) and heated by microwave for 30 min, after routine deparaffinization and rehydration. The endogen peroxidase activity was inhibited with 3% H_2_O_2_ for 10 min and nonspecific binding was blocked with 10% normal goat serum for 1 h. All sections were incubated with primary antibodies for DDx4/MVH (1:200, ab13840) for 24 h at 4 °C and then placed for 45 min at room temperature. Horseradish peroxidase (HRP) combined secondary antibodies were added for 30 min, and visualization of the antigens was achieved by diaminobenzidine (DAB) staining. The slides were counterstained with hematoxylin, then dehydrated and mounted finally. The sections were rinsed with PBS three times for 5 min between each step. At the same time, a negative control was treated by substituting phosphate buffered saline (PBS) for primary antibodies.

### 4.4. Western Blot

Mouse ovaries were placed in liquid nitrogen and then extracted by RIPA lysis buffer (P0013B, Beyotime Institute of Biotechnology, ShangHai, China) with protease inhibitor PMSF (ST506, Beyotime Institute of Biotechnology). Protein content was determined using a BCA Protein Assay Kit (P0012, Beyotime Institute of Biotechnology). After separation by electrophoresis, proteins were electronically transferred to polyvinylidenefluoride (PVDF) membranes. Membranes were then blocked in 5% skimmed milk-PBST (PBS containing 0.1% Tween 20) for 1 h and incubated overnight at 4 °C with specific antibodies. Antibodies against mTOR (2983), p-mTOR (S2448) (5536), p70S6 (2708), p-p70S6 (T389) (9234), rpS6 (2217), p-rpS6 (S240/244) (5364), eIF4B (3592), p-eIF4B (S422) (3591), APAF-1 (8969), cleaved caspase 3 (9664), and caspase 3 (9662), GAPDH, and β-tubulin (2128) were all rabbit monoclonalantibodies purchased from Cell Signaling Technology (CST, Danvers, MA, USA). Horseradish peroxidase (HRP)-conjugated goat anti-rabbit IgG (7074, CST) was used to detect proteins through enhanced chemiluminescence (15049, Millipore, Danvers, MA, USA). GAPDH or β-tubulin expression was measured to verify equal loading.

### 4.5. TUNEL Assay

Follicle apoptosis in ovarian tissue sections was identified using an *in situ* cell death detection kit (Roche Company, Basel, Switzerland). All the slides were incubated in a freshly prepared 0.1% Triton X-100 permeabilization solution with 0.1% citrate buffer for 8 min, after deparaffinization and rehydration. Next, the slides were fully immersed in Tris–HCl, 0.1 M pH 7.5, containing 20% normal bovine serum and 3% BSA for 30 min at room temperature. Then, 50 μL of TUNEL reaction mixture were added on the section and the slides were incubated for 1 h at 37°C. Finally, the slides were rinsed and the sections were evaluated under a fluorescence microscope.

## 5. Conculsions

The results of the current study suggested that both the PI3K/Akt/mTOR and the classical apoptosis signaling pathways were hyperactivated in CTX-treated mice, which induced primordial follicle loss and growing follicle apoptosis, ultimately damaging fertility. Activation of mTORC1 stimulates angiogenesis and inhibits autophagy, and these processes are all essential in tumorigenesis [20]. Thus, any medication that can prevent hyperactivation of the PI3K/Akt/mTOR signaling pathway may have the potential to act as an ovarian-protective agent and may be administered to female cancer patients for fertility preservation. Additionally, deregulation of the PI3K/Akt/mTOR signaling pathway is also associated with human cancer therapy.

## References

[1] Loren A.W., Mangu P.B., Beck L.N., Brennan L., Magdalinski A.J., Partridge A.H., Quinn G., Wallace W.H., Oktay K. (2013). Fertility preservation for patients with cancer: American Society of Clinical Oncology clinical practice guideline update. J. Clin. Oncol..

[2] Meirow D., Dor J., Kaufman B., Shrim A., Rabinovici J., Schiff E., Raanani H., Levron J., Fridman E. (2007). Gonadal dysfunction and fertility problems in cancer survivors. Acta. Oncol..

[3] Stroud J.S., Mutch D., Rader J., Powell M., Thaker P.H., Grigsby P.W. (2009). Effects of cancer treatment on ovarian function. Fertil. Steril..

[4] Salama M., Winkler K., Murach K.F., Seeber B., Ziehr S.C., Wildt L. (2013). Female fertility loss and preservation: Threats and opportunities. Ann. Oncol..

[5] Meirow D., Dor J., Kaufman B., Shrim A., Rabinovici J., Schiff E., Raanani H., Levron J., Fridman E. (2007). Cortical fibrosis and blood-vessels damage in human ovaries exposed to chemotherapy. Potential mechanisms of ovarian injury. Hum. Reprod..

[6] Meirow D., Biederman H., Anderson R.A., Wallace W.H. (2010). Toxicity of chemotherapy and radiation on female reproduction. Clin. Obstet. Gynecol..

[7] Burnell M., Levine M.N., Chapman J.A., Bramwell V., Gelmon K., Walley B., Vandenberg T., Chalchal H., Albain K.S., Perez E.A. (2010). Cyclophosphamide, epirubicin, and Fluorouracil *versus* dose-dense epirubicin and cyclophosphamide followed by Paclitaxel *versus* Doxorubicin and cyclophosphamide followed by Paclitaxel in node-positive or high-risk node-negative breast cancer. J. Clin. Oncol..

[8] Behringer K., Mueller H., Goergen H., Thielen I., Eibl A.D., Stumpf V., Wessels C., Wiehlpütz M., Rosenbrock J., Halbsguth T. (2013). Gonadal function and fertility in survivors after Hodgkin lymphoma treatment within the German Hodgkin Study Group HD13 to HD15 trials. J. Clin. Oncol..

[9] Devi H.P., Mazumder P.B. (2016). Methanolicextract of curcuma caesiaRoxb. Prevents the toxicity caused by cyclophosphamide to bone marrow cells, liver and kidney of mice. Pharmacogn. Res..

[10] Song Y., Zhang C., Wang C., Zhao L., Wang Z., Dai Z., Lin S., Kang H., Ma X. (2016). Ferulicacid against cyclophosphamide-induced Heart toxicity in mice by inhibiting NF-κB pathway. Evid.-Based. Complement. Altern. Med..

[11] Li F., Turan V., Lierman S., Cuvelier C., de Sutter P., Oktay K. (2014). Sphingosine-1-phosphate prevents chemotherapy-induced human primordial follicle death. Hum. Reprod..

[12] Blumenfeld Z. (2002). Preservation of fertility and ovarian function and minimalization of chemotherapy associated gonadotoxicity and premature ovarian failure: The role of inhibin-A and -B as markers. Mol. Cell. Endocrinol..

[13] Adhikari D. (2013). *In vitro* activation of dormant follicles for fertility preservation. Adv. Exp. Med. Biol..

[14] Reddy P., Zheng W., Liu K. (2010). Mechanisms maintaining the dormancy and survival of mammalian primordial follicles. Trends Endocrinol. Metab..

[15] Reddy P., Liu L., Adhikari D., Jagarlamudi K., Rajareddy S., Shen Y., Du C., Tang W., Hämäläinen T., Peng S.L. (2008). Oocyte-specific deletion of *Pten* causes premature activation of the primordial follicle pool. Science.

[16] Adhikari D., Risal S., Liu K., Shen Y. (2013). Pharmacological inhibition of mTORC1 prevents over-activation of the primordial follicle pool in response to elevated PI3K signaling. PLoS ONE.

[17] Adhikari D., Zheng W., Shen Y., Gorre N., Hämäläinen T., Cooney A.J., Huhtaniemi I., Lan Z.J., Liu K. (2010). Tsc/mTORC1 signaling in oocytes governs the quiescence and activation of primordial follicles. Hum. Mol. Genet..

[18] Tong Y., Li F., Lu Y., Cao Y., Gao J., Liu J. (2013). Rapamycin-sensitive mTORC1 signaling is involved in physiological primordial follicle activation in mouse ovary. Mol. Reprod. Dev..

[19] El-Serafi I., Afsharian P., Moshfegh A., Hassan M., Terelius Y. (2015). Cytochrome P450 oxidoreductase influences CYP2B6 activity in cyclophosphamide bioactivation. PLoS ONE.

[20] Van Veelen W., Korsse S.E., van de Laar L., Peppelenbosch M.P. (2011). The long and winding road to rational treatment of cancer associated with LKB1/AMPK/TSC/mTORC1 signaling. Oncogene.

